# Arthropods Associated with Invasive *Frangula alnus* (Rosales: Rhamnaceae): Implications for Invasive Plant and Insect Management

**DOI:** 10.3390/insects14120913

**Published:** 2023-11-28

**Authors:** Jennifer Greenleaf, Ida Holásková, Elizabeth Rowen, Michael Gutensohn, Richard Turcotte, Yong-Lak Park

**Affiliations:** 1 Division of Plant and Soil Sciences, West Virginia University, Morgantown, WV 26506, USA; jennifers.greenleaf@gmail.com (J.G.); elizabeth.rowen@mail.wvu.edu (E.R.); michael.gutensohn@mail.wvu.edu (M.G.); richard.m.turcotte@usda.gov (R.T.); 2 Office of Statistics and Data Analytics, West Virginia Agricultural and Forestry Experiment Station, Davis College of Agriculture, Natural Resources and Design, West Virginia University, Morgantown, WV 26506, USA; ida.holaskova@mail.wvu.edu; 3 State, Private and Tribal Forestry, USDA Forest Service, Morgantown, WV 26505, USA

**Keywords:** glossy buckthorn, *Frangula alnus*, insect, richness, diversity, community, *Drosophila suzukii*, volatile organic compounds

## Abstract

**Simple Summary:**

Invasive plants pose a significant threat to native ecosystems, and understanding their interactions with local arthropod communities is crucial for effective management strategies. This study aimed to identify arthropod species associated with glossy buckthorn (*Frangula alnus*), an invasive plant originating from Europe and colonizing North America. Over two years, arthropod samples were systematically collected from 16 different plots on five collection dates. The collected arthropods were identified, and their abundance, richness, and diversity were measured. Additionally, the study documented the arthropods emerging from *F. alnus* fruits and volatiles emitted by *F. alnus*. The results revealed that the dominant insect orders were true bugs (Hemiptera, 39.8%) and flies (Diptera, 22.3%). The dominant species was *Psylla carpinicola* (Hemiptera: Psyllidae), followed by *Drosophila suzukii* (Diptera: Drosophilidae); *D. suzukii* utilized *F. alnus* fruits for reproduction. Furthermore, the study noted that the abundance, richness, and diversity of arthropod orders were influenced by the phenology of *F. alnus*. This pioneering research provides valuable insights into the arthropod communities associated with *F. alnus* in North America, offering a foundation for the development of effective management strategies to control this invasive plant species.

**Abstract:**

The invasive shrub glossy buckthorn (*Frangula alnus*) has been progressively colonizing the Northeastern United States and Southeastern Canada for more than a century. To determine the dominant arthropod orders and species associated with *F. alnus*, field surveys were conducted for two years across 16 plots within the Allegheny National Forest, Pennsylvania, USA. Statistical analyses were employed to assess the impact of seasonal variation on insect order richness and diversity. The comprehensive arthropod collection yielded 2845 insects and arachnids, with hemipterans comprising the majority (39.8%), followed by dipterans (22.3%) and arachnids (15.5%). Notably, 16.2% of the hemipterans collected were in the immature stages, indicating *F. alnus* as a host for development. The two dominant insect species of *F. alnus* were *Psylla carpinicola* (Hemiptera: Psyllidae) and *Drosophila suzukii* (Diptera: Drosophilidae); *D. suzukii* utilized *F. alnus* fruits for reproduction. Species richness and diversity exhibited significant variations depending on the phenology of *F. alnus*. The profiles of volatile compounds emitted from the leaves and flowers of *F. alnus* were analyzed to identify factors that potentially contribute to the attraction of herbivores and pollinators. The results of our study will advance the development of novel *F. alnus* management strategies leveraging the insects associated with this invasive species.

## 1. Introduction

Among the several species of *Frangula* (Rosales: Rhamnaceae) in North America, glossy buckthorn (*Frangula alnus*) is an invasive European shrub that has been spreading throughout the northeastern portion of the United States and Southeastern Canada [1]. It is an aggressive shrub as it forms dense monospecific patches in disturbed areas, along roads, and throughout fields and clearings [2]. In its native European range, *F. alnus* was utilized as a source of superior charcoal for gunpowder production, as a laxative, and as a sap-green dye [3]. In the late 1800s, it was introduced to Canada as an ornamental and was first collected outside of cultivation in London, Canada, in 1898. By 1970, it had spread to areas up to 150 km away from its original introduction [4]. Currently, *F. alnus* has been reported in 29 US states [5].

As is typical of invasive species, *F. alnus* has several factors contributing to its success in North America. It appears to lack the natural herbivores that it experienced in Europe, potentially not being exposed to the necessary pest pressure for control in North America. *Zygina suavis* (Hemiptera: Cicadellidae) and *Gonopteryx rhamni* (Lepidoptera: Pieridae) are both herbivorous insects known to be directly associated with *F. alnus* in Europe [6,7]. These insects can limit the growth and reproduction of *F. alnus* in its native habitats, but these inhibitory and specialist species are not known to exist in North America, highlighting the importance of identifying inhibitory species already present within North America. Light feeding damage due to deer browsing was also reported, but the damage did not affect invasion [8]. In addition to occupying a natural enemy-free landscape, the ability of *F. alnus* to flourish in a variety of soil conditions [3] increases its suitable range and could, in turn, increase connectivity between potential open habitats. This sun-loving plant also puts out new leaves before other deciduous trees and retains its leaves longer into the fall than other deciduous trees [9]. This results in a longer growing season, allowing the invasive shrub to monopolize soil nutrients and sunlight, crowding out native undergrowth with its vigorous growth.

In Europe, multiple projects have previously compiled lists of European insects associated with *F. alnus*. A study in Southern Spain [10] reported 47 insect species utilizing the flowers and suspected that 21 of them were likely to pollinate the flowers. Dipterans were the dominant insect group in terms of both the number of species interacting with the flowers and the number of individuals. To identify candidate natural enemies that could be introduced to North America as a weed control solution, Gassmann et al. [6] collected 1000 insect samples from 99 sites containing *F. alnus* and common buckthorn, *Rhamnus cathartica* (Rosales: Rhamnaceae), in Europe. They discovered eight insect species associated with *F. alnus*, with *Z. suavis* being exclusive to *F. alnus*. Brändle and Brandl [11] found that 91 phytophagous insect species were associated with the combined genera *Frangula* and *Rhamnus* in Germany, 29 of which were specialists. Simandl [12] specifically investigated wood-boring beetles associated with *F. alnus* in the South Bohemian region and found a total of 13 associated species. Moreover, the specialist *G. rhamni* is known to utilize *F. alnus* in Europe [6]. There are few mentions in the literature of individual insect species found to be associated with *F. alnus* in North America, such as the soybean aphid, *Aphis glycines* (Hemiptera: Aphididae), which utilizes *F. alnus* for overwintering [13]. Although these earlier studies have profiled insects associated with *F. alnus* in Europe, a similar comprehensive compilation has not been conducted in North America. 

Understanding how an invasive species interacts with ecosystems in its invaded range can lead to improvements in the management of the species. Differences in soil conditions, climate, pollinators, and herbivores in the invaded range compared to the native range could play a key role in the success or failure of an invasive plant. While the herbivorous arthropods that coevolved with *F. alnus* in Europe do not exist in North America, it is still possible that North American insects could accept *F. alnus* as a host plant. Insect-mediated pollination and the resulting fruit set are decisive factors in the successful spread of an invasive plant species. Thus, surveying insect communities on *F. alnus* will help to identify key insect species that could be associated with *F. alnus*, including those that utilize this shrub as a pest reservoir. Another important aspect of understanding the interaction of an invasive plant species with its biotic environment, including its interactions with insects, is detailed knowledge of the specialized plant metabolites that it produces. *F. alnus* is well known for the formation of anthraquinones, including the abundant emodin [14], which have antifeeding, laxative, antimicrobial, and allelopathic activity [15]. In contrast, very little is yet known about the volatile organic compounds (VOCs) that are emitted from leaves and flowers of *F. alnus*, although they are generally known to be involved in the interaction with herbivores and the attraction of pollinators [16]. The interaction of plants with insect herbivores is influenced by VOCs, a group of specialized metabolites that are emitted from vegetative plant tissues into the surrounding atmosphere. While herbivores utilize some of these VOCs to localize suitable host plants, VOC emission is also an essential part of the plant defense against herbivores, either directly by acting as a repellent or indirectly by attracting predators and parasitoids [17]. Moreover, the emission of defense-related plant VOCs is often induced by herbivory through plant tissue being wounded or exposed to herbivore-derived molecular patterns such as oral saliva [18].

The goal of this study was to determine the associations between North American insects and *F. alnus* to provide a foundation for potential management strategies. This research specifically aimed to determine (1) the dominant insect orders and species found on *F. alnus*, (2) the changes in their species richness and diversity throughout the growing season, (3) insects that utilize the fruits of *F. alnus*, and (4) volatile organic compounds emitted from flowers and leaves of *F. alnus* that contribute to the interaction with insects.

## 2. Materials and Methods

### 2.1. Study Site 

**Figure 1:**
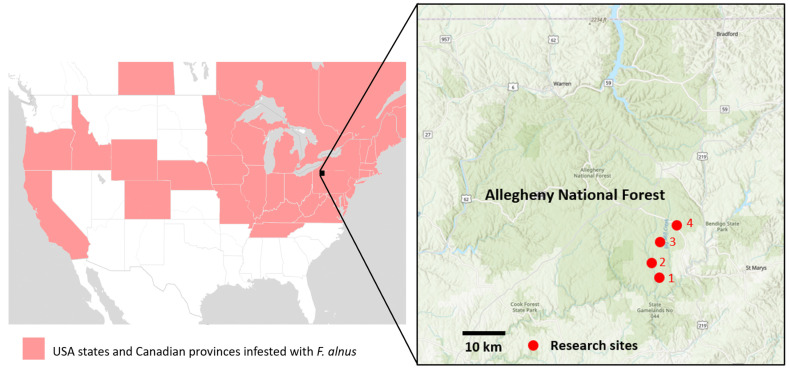
The locations of four research sites (Sites 1–4 above) near the town of Ridgway, Pennsylvania. Each site had four plots and arthropod sampling was conducted at five different times in 2021 and 2022. Distribution of *F. alnus* as of 2023 was adopted from EDDMaps (https://www.eddmaps.org access on 10 November 2023).

This research was conducted in the Allegheny National Forest near the borough of Ridgway, PA, USA. Four 2.5-ha sites (sites 1–4) infested with *F. alnus* were selected, and, within each site, four 1-m^2^ plots of *F. alnus* (i.e., a total of 16 plots) were established. The geocoordinates of the four sites were 41.43704, −78.79137 for Site 1; 41.45674, −78.80247 for Site 2; 41.47875, −78.77288 for Site 3; and 41.485216, −78.741848 for Site 4 (Figure 1). Sites 1 and 2 are primarily deciduous forests with intersecting unpaved forest roads. Site 3 is more varied, with a powerline, deciduous trees, grass fields, and a stream. Site 4 consists of mesic deciduous forests, paved and unpaved roads, wet areas, and a patch of conifers (Figure 1). Our plot placements and the selection of individual *F. alnus* for sampling were strategically determined to target areas where *F. alnus* populations existed in isolation from other plant species, ensuring a focused investigation into the ecological interactions associated with this invasive shrub. 

### 2.2. Arthropod Sampling

Arthropods were collected from *F. alnus* on 16 plots using an aerial insect net. The insect net, with a diameter of 30 cm, was swept back and forth 10 times in a zig-zag pattern deeply and harshly into the branches of *F. alnus* to cover an area of 1 m in width by 2.5 m in height for each sample. All collections took place between noon and 4 p.m. on sunny or partly cloudy days, with wind speeds below 25 kph. This was replicated on five dates (4 August 2021; 6 September 2021; 19 May 2022; 30 June 2022; and 22 July 2022), resulting in a total of 80 samples (i.e., 16 plots on five sample dates). In addition to insect net sampling, we directly observed and recorded any insects feeding and visiting flowers and fruits. 

The collected insects were brought to the lab and sorted to order and then to morphospecies by using a Dino-Lite Digital Microscope (Dunwell Tech., Inc. dba Dino-Lite Microscope, Torrance, CA, USA). The most common morphospecies were identified to species.

### 2.3. Fruit Sampling

Ripe *F. alnus* fruits were collected directly from the live branches of *F. alnus* at each of the 16 sites on three collection dates: 6 September 2021; 22 July 2022; and 10 August 2022. An average of 14 fruits were randomly selected and collected per plot on each date. The collected fruits were then placed in Solo P550N 5.5-oz plastic cups (Solo Cup Company, Lake Forest, IL, USA) with mesh placed over the top to prevent the escape of emerging insects. We collected an additional 434 *F. alnus* fruits at four different ripening stages based on color (i.e., green, greenish red, red, and black) in August and September 2023 to determine which fruit ripening stage that the insects used to lay eggs, with black representing fully ripened fruit. These cups were kept in the laboratory at ~21 °C, and the insect emergence from them was recorded. Each insect that emerged from these fruits was identified to the species level and sexed.

### 2.4. Collection and Analysis of Volatile Organic Compounds

Branches from *F. alnus* were collected in the Allegheny National Forest and placed into a water-filled container for transport. Volatiles emitted from *F. alnus* leaves were collected using a closed-loop stripping method as described by Wang et al. [19,20]. Five leaves were cut from freshly harvested branches for each volatile collection and supplemented with 10% (*w*/*v*) sucrose solution. In addition, leaves were also wounded using an array of needles as described by Gutensohn et al. [21]. Headspace collections from detached unwounded and wounded leaves were performed for 24 h using Porapak-Q traps (Volatile Collection Trap LLC, Gainesville, FL, USA), and collected volatile compounds were subsequently eluted with dichloromethane. Moreover, 3.33 µg of naphthalene was added as an internal standard.

Samples from headspace collections were analyzed by combined gas chromatography/mass spectrometry (GC/MS) using a TRACE 1310 gas chromatograph system linked to a TSQ 8000 triple quadrupole mass spectrometer (Thermo Fisher Scientific, Waltham, MA, USA), as described by Wang et al. [19,20]. Individual compounds were identified using the Xcalibur 2.2 SP1.48 software (Thermo Fisher Scientific) by comparing their mass spectra with those deposited in the NIST/EPA/NIH Mass Spectral Library (NIST11) (National Institute of Standards and Technology NIST, Scientific Instrument Services, Inc., Ringoes, NJ, USA; https://chemdata.nist.gov/mass-spc/ms-search/ accessed on 10 November 2023).

For the analysis of floral volatile emissions, ten freshly sampled *F. alnus* flowers, either before or after anthesis, were placed in 2-mL glass vials and closed with an airtight screwcap. Floral volatiles were collected from the headspace via stir bar sorptive extraction (SBSE) by attaching a magnetic Twister^®^ coated with polydimethylsiloxane (PDMS) (Gerstel, Mülheim, Germany) to the inside of the glass vial with an additional small magnet (D401-N52, K&J Magnetics, Pipersville, PA, USA). After 24 h, the Twisters were removed from the glass vials, and collected volatiles were analyzed by GC/MS utilizing a coupled gas chromatograph (Agilent Technologies 7890B series)/mass spectrometer (Agilent Technologies 5977B Inert Plus MSD Turbo EI, Santa Clara, CA, USA) equipped with a thermal desorption unit (TDU) and a cooled injection system (CIS 4C) (Gerstel, Mülheim, Germany). 

### 2.5. Data Analysis

Arthropods collected in this study were grouped into eight categories: Arachnida, Coleoptera, Diptera, Hemiptera, Hymenoptera, Lepidoptera, Psocodea, and Others (all other minor insect orders). To determine which arthropod orders and morphospecies were dominant, the numbers of occurrences of each order and morphospecies were summed up and then ranked. The five dominant morphospecies were identified to species.

The arthropod abundance and arthropod diversity of each order over the five collection dates were analyzed using repeated-measures ANOVA followed by Tukey–Kramer comparisons of least squares means in PROC GLIMMIX of SAS. To measure the arthropod abundance, the statistical distribution of data was examined by the Shapiro–Wilk W test for a lack of normality. We found a lack of normality in the dataset and the data fit the Poisson error distribution, and thus we used PROC GLIMMIX with Poisson distribution as a log link function (SAS^®^, Version 9.4, SAS Institute Inc., Cary, NC, USA), after a constant 1 was added to the raw data. The model included the effects of order and time (i.e., the number of weeks since the beginning as a categorical variable type), and their interaction, with time as a random effect (repeated) and individual plots as subjects. A slicing method of multiple comparisons with Tukey–Kramer adjustment was employed. It enabled, in the case of an existing interaction of two effects, multiple comparisons among time points within each order (e.g., Hemiptera in August 2021 vs. Hemiptera in September 2021) or comparisons among the orders at each of the five sampling collections (e.g., Arachnida vs. Diptera in May 2022). Slicing prevents confounding and nonlogical comparisons (e.g., Arachnida in August 2021 vs. Lepidoptera in May 2022).

Arthropod richness was calculated by summing the number of morphospecies for each of the 16 plots on each of the five dates by using R software 9, version 4.1.2 [22]. The effect of the collection date (categorical) on richness was analyzed using a repeated-measures generalized linear mixed model with a negative binomial error distribution using the package glmmTMB in R [23]. To account for repeated measurements at each site, the site and plot nested in the site were included as random intercepts. Violations of assumptions were tested using the package DHARMa. The Levene test was used to check for homoscedasticity. Collection dates were compared using a post hoc Tukey–Kramer multiple comparison analysis (package emmeans) [24]. 

Arthropod diversity was measured using the inverse Simpson’s diversity index (*D_InvSimpson_*). The diversity index for each of the 16 plots on each of the five dates (for a total of 80 calculations) was calculated using the following equation:

$$D_{InvSimpson}=\frac{1}{\sum_{i=1}^{s}p_{i}^{2}}$$

where *s* represents the number of unique morphospecies present in the given plot and date, *i* represents each morphospecies, and *P_i_* represents the proportion of abundance of morphospecies *i* within the given plot and date. *P_i_* = *N_i_*/*N*, where *N* represents total collection abundance and *N_i_* represents the abundance within morphospecies *i*. It may be noted that it is common practice to divide one by the Simpson’s diversity index to obtain an inverse index, to ensure that higher indices represent higher diversity [25]. To meet the assumptions of normality of distribution for repeated-measures ANOVA, the inverse Simpson’s diversity index was transformed by taking the log.

## 3. Results

### 3.1. Summary of Arthropod Survey

Of the 2845 arthropod individuals collected in the net sweeping of *F. alnus*, 1131 (39.8%) were hemipterans, 635 (22.3%) were dipterans, and 442 (15.5%) were arachnids (Table 1). Hemipterans were dominant in the total collection of individuals and on the August 2021, June 2022, and July 2022 collection dates. In September 2021 and May 2022, dipterans had the highest abundance.

### 3.2. Major Arthropod Species

**Figure 2:**
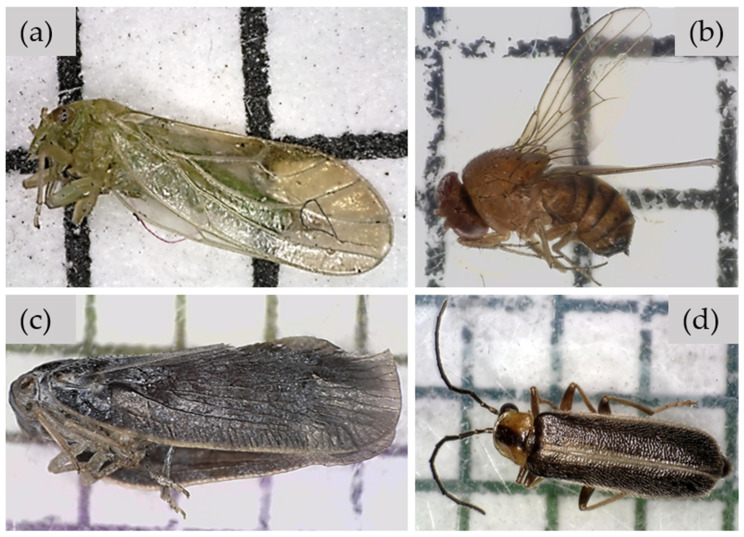
The top four dominant morphospecies collected by net sweeping of *F. alnus*: *Psylla carpinicola* (Hemiptera: Psyllidae) (**a**), *Drosophila suzukii* (Diptera: Drosophilidae) (**b**), *Metcalfa pruinosa* (Hemiptera: Flatidae) (**c**), and *Rhagonycha recta* (Coleoptera: Cantharidae) (**d**). One square in the background of each image is 2 mm by 2 mm.

There were 563 morphospecies found in this study. The dominant insect species was *Psylla carpinicola* (Hemiptera: Psyllidae), which constituted 22.0% of the individual insects collected in the net sweeps. A total of 137 *P. carpinicola* were caught in August 2021, 290 in June 2022, and 180 in July 2022. Only 18 were collected in September 2021 and none in May 2022. The second dominant insect species captured in the net sweeping was the spotted-wing drosophila *Drosophila suzukii* (Diptera: Drosophilidae), all of which were captured only in September 2021. A total of 135 *D. suzukii* were collected in this study: 48 males and 87 females. The third dominant species was the citrus flatid *Metcalfa pruinosa* (Hemiptera: Flatidae), with 59 adults and 70 nymphs. The fourth dominant species collected via net sweeping was the soldier beetle *Rhagonycha recta* (Coleoptera: Cantharidae) (53 individuals) (Figure 2). 

In addition to insect net sampling, we directly observed insects utilizing *F. alnus*. In August 2022 and 2023, we frequently observed two stink bug species (the brown stink bug *Euschistus servus* and the green stink bug *Chinavia halaris*) feeding on the ripe fruits of *F. alnus*. These stink bug species were collected in the net sweeping collection as well. In addition, a lepidopteran caterpillar was found to have consumed much of the top leaves of an individual *F. alnus* sapling. The caterpillar was collected to rear it into an adult for identification, but, within a few days, the caterpillar died of a fungal infection. Despite our systematic effort to observe pollinators visiting *F. alnus* flowers, we only observed a relatively limited abundance and number of species of insects visiting the flowers. The major insects visiting flowers were the common eastern bumblebee *Bombus impatiens* (Hymenoptera: Apidae), eastern yellowjacket *Vespula maculifrons* (Hymenoptera: Vespidae), pure green sweat bee *Augochlora pura* (Hymenoptera: Halictidae), and *R. recta* (Figure 2d). Our survey also revealed that there are numerous natural enemies indirectly associated with *F. alnus*, including parasitic wasps (i.e., Chalcididae, Ichneumonidae, and Braconidae) (Hymenoptera) and arachnids, which constituted 15.5% of the net sweep collection.

Although the abundance of lepidopteran larvae was not very high, they fed on *F. alnus* foliage. The lepidopteran larvae captured from *F. alnus* by net sweeping in this study belonged to the insect families of Erebidae, Gelechiidae, Geometridae, Noctuidae, Saturniidae, Crambidae, and Psychidae. Notably, larvae of Geometridae were the most abundant among the families, although only 28 individual larvae were collected throughout the study period. Additional lepidopteran species identified included the fall webworm *Hyphantria cunea* (Lepidoptera: Erebidae), fluid arches *Morrisonia latex* (Lepidoptera: Noctuidae), and spotted beet webworm *Hymenia perspectalis* (Lepidoptera: Crambidae).

### 3.3. Seasonal Changes in Arthropod Community on F. alnus

**Figure 3:**
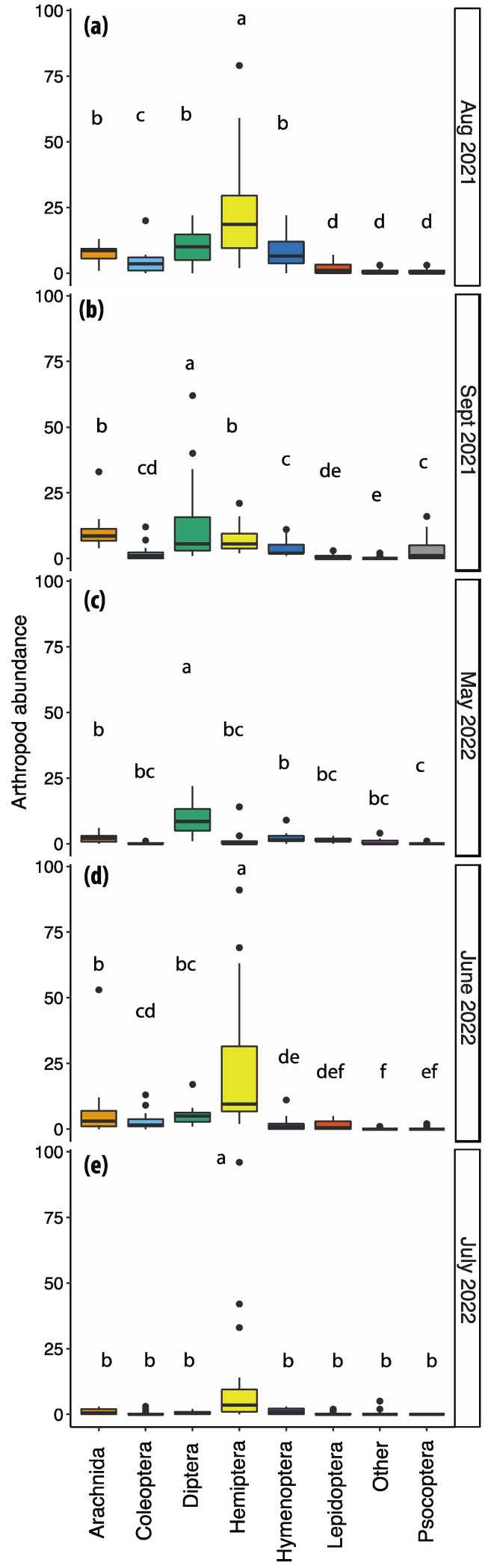
Abundance of arthropods within four sites, collected on each net sweeping date, separated by order grouping. Collection dates are separated in each panel (**a**–**e**). Bars sharing a letter (above the boxplots) are not significantly different (Tukey HSD, α = 0.05). Boxplots show the median (horizontal line), the first and third quartile (box), and 1.5 times the interquartile range (whisker).

Our analysis of insect and arachnid abundance at different sampling times during the annual growing season of *F. alnus* detected a significant effect of the sampling month (F = 11.89, d.f. = 4, 75, *p* < 0.0001) and order (F = 137.26, d.f. = 7, 525, *p* < 0.0001), as well as an interaction of order and sampling month (F = 20.46, d.f. = 28, 525, *p* < 0.0001). Specifically, the numbers of insects and arachnids were significantly higher in August and September 2021 compared to June and July 2022. Hemipterans were significantly more abundant in August 2021, June, and July 2022 than all other orders (Figure 3), which was primarily due to the abundance of *P. carpinicola* as well as *M. pruinosa*. However, Hemipteran abundance was significantly lower than that of Diptera in May 2022 (Figure 3), when *F. alnus* had the lowest leaf coverage of all five survey dates. Of the 250 hemipterans collected in August 2021, 137 were *P. carpinicola* and 54 were *M. pruinosa*. Of the 380 hemipterans collected in June 2022, 290 were *P. carpinicola*. Dipterans were the most abundant in August, September, and May but lower in June and July.

**Figure 4:**
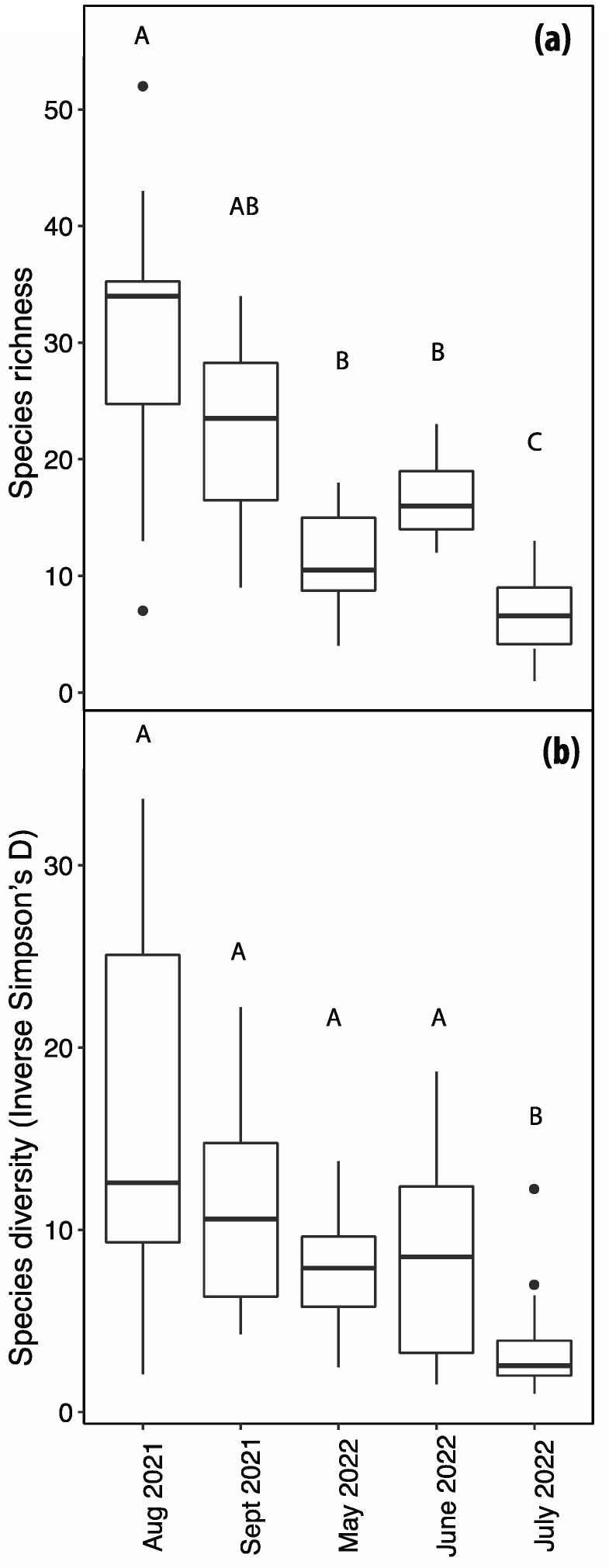
Species richness (**a**) and diversity (**b**) amongst the five dates. Bars sharing a letter (above the boxplots) are not significantly different. Boxplots show the median (horizontal line), the first and third quartile (box), and 1.5 times the interquartile range (whisker).

Species richness varied amongst the collection dates (χ^2^ = 88.9, d.f. = 4, *p* < 0.0001). A Tukey–Kramer multiple comparisons analysis indicated that the species richness was significantly higher in August 2021 than in May, June, and July 2022 (Figure 4a). Richness differed most between August 2021 and July 2022. In addition to richness, species diversity varied among the collection dates (F = 9.69, d.f. = 4, 75, *p* < 0.0001) and diversity was significantly lower in July 2022 than at the other sample dates (Figure 4b). 

### 3.4. Insect Emergence from F. alnus Fruits

All insects that emerged from the collected *F. alnus* fruits were *D. suzukii*. From the 685 fruits collected, 216 adult *D. suzukii* emerged. Of those emerged *D. suzukii* 105 were male, and 111 were female, representing a 1 male to 1.06 female sex ratio. While, at one of the three collection dates (July 2022) no insects emerged from the 160 collected fruits, in August 2022, 51.5% of the emerging *D. suzukii* were male, and in September 2021, 43.8% were male. In the net sweeping collection of insects from *F. alnus*, *D. suzukii* was also one of the most abundant morphospecies. However, the sex ratio of *D. suzukii* captured during net sweeping was significantly different, with 64.4% female and 35.6% male flies, representing a 1 male to 1.81 female ratio. Remarkably, we found that adult *D. suzukii* only emerged from the black, fully ripened *F. alnus* fruits, but no *D. suzukii* adults emerged from fruits harvested before ripening, suggesting that the oviposition and development of *D. suzukii* occur at later stages of *F. alnus* fruit ripening. 

### 3.5. Volatile Profiles of F. alnus Leaves and Flowers

**Figure 5:**
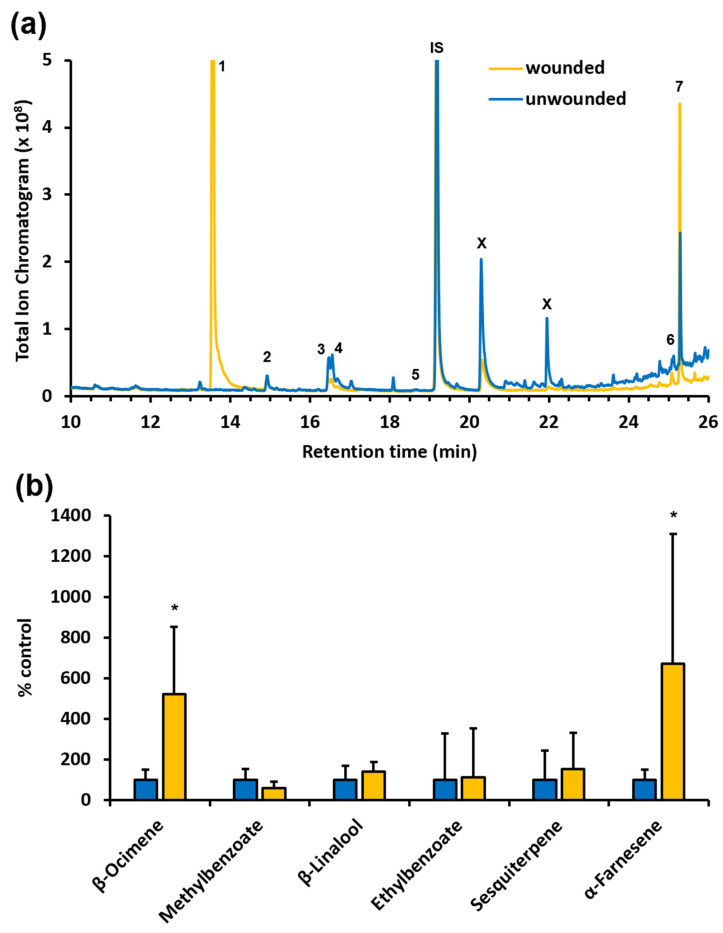
Characterization of the profile of volatile organic compounds emitted from unwounded and wounded *F. alnus* leaves. (**a**) Volatiles were analyzed by GC/MS, and total ion chromatograms are shown for unwounded and wounded leaves. The following compounds were identified by the comparison of mass spectra with the NIST library: 1, 3-hexenyl acetate; 2, β-ocimene; 3, methyl benzoate; 4, β-linalool; 5, ethyl benzoate; 6, unknown sesquiterpene; 7, α-farnesene; X, contamination; IS, internal standard. (**b**) Abundance of individual volatile compounds emitted from wounded and unwounded leaves (yellow and blue bars, respectively). Values for abundance represent means ± SE (*n* ≥ 4 biological replicates). Quantities of compounds emitted from wounded leaves are presented as a percentage of the corresponding value in unwounded leaves, set as 100%. Asterisks indicate statistically significant differences (* *p* < 0.03 by Student’s *t*-test).

To better understand the interaction of arthropods with *F. alnus* in its invasive North American range, we collected the volatiles emitted from unwounded and wounded *F. alnus* leaves, as well as from *F. alnus* flowers, and analyzed these by gas chromatography/mass spectrometry. This analysis (Figure 5a) showed that *F. alnus* leaves are not very strongly scented and only emit a limited number of VOCs, including the monoterpenes β-ocimene and β-linalool, an unknown sesquiterpene, and α-farnesene, as well as the aromatic compounds methyl benzoate and ethyl benzoate. When we analyzed the volatile profile of wounded *F. alnus* leaves (Figure 5a), we found, as expected, a strong emission of the green leaf volatile 3-hexenylacetate and occasionally some 3-hexen-1-ol. In addition, the emission of β-ocimene and α-farnesene was increased upon wounding of leaves (Figure 5b), while that of methyl benzoate, ethyl benzoate, β-linalool, and the unknown sesquiterpene remained unchanged compared to unwounded leaves. 

**Figure 6:**
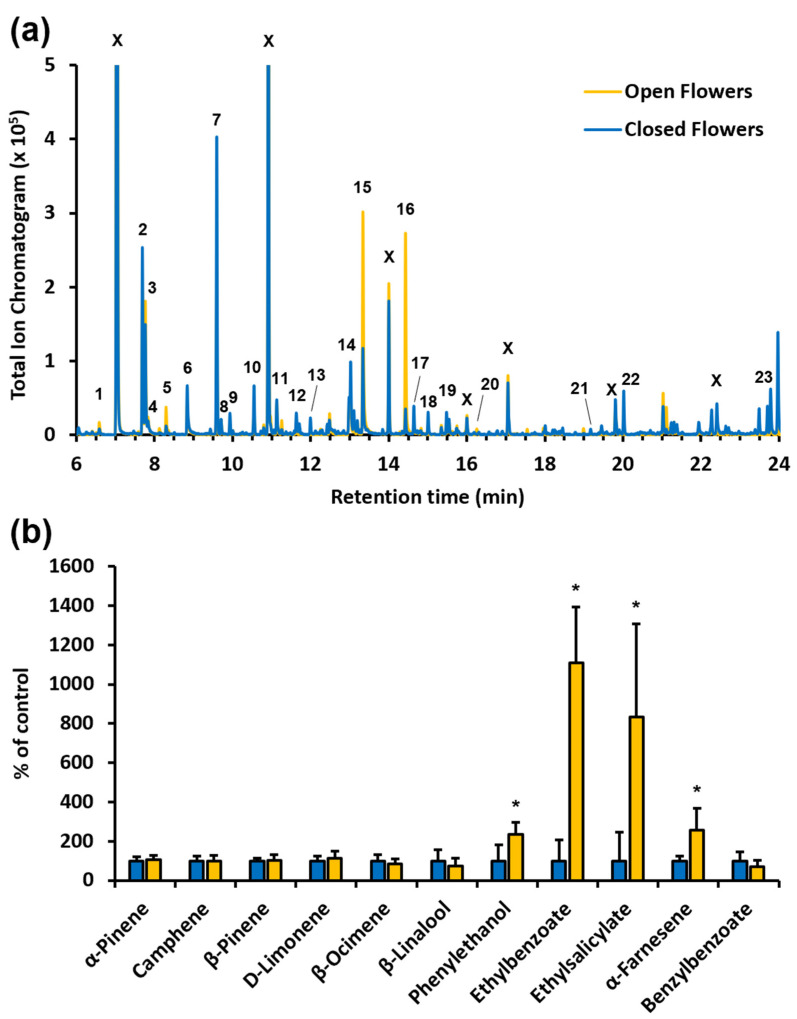
Characterization of the profile of volatile organic compounds emitted from closed and open *F. alnus* flowers. (**a**) Volatiles were analyzed by GC/MS, and total ion chromatograms are shown for closed and open flowers. The following compounds were identified by the comparison of mass spectra with the NIST library: 1, ethyl butyrate; 2, ethyl 2-methyl butyrate; 3, ethyl 3-methyl butyrate; 4, 2-methyl butyric acid; 5, isoamylacetate; 6, oxime methoxy phenyl; 7, α-pinene; 8, ethyl tiglate; 9, camphene; 10, β-pinene; 11, 3-hexenyl acetate; 12, D-limonene; 13, β-ocimene; 14, β-linalool; 15, phenylethanol; 16, ethyl benzoate; 17, 3-hexenyl isobutyrate; 18, decanal; 19, 3-hexenyl isovalerate; 20, ethyl salicylate; 21, isoeugenol; 22, α-farnesene; 23, benzylbenzoate; X, contamination from the PDMS-coated Twister. (**b**) Abundance of individual volatile compounds emitted from open and closed flowers (yellow and blue bars, respectively). Values for abundance represent means ± SE (*n* = 5 biological replicates). Quantities of compounds emitted from open flowers are presented as a percentage of the corresponding value in closed flowers, set as 100%. Asterisks indicate statistically significant differences (* *p* < 0.02 by Student’s *t*-test).

Our analysis revealed that *F. alnus* flowers (Figure 6a) from plants in the Allegheny National Forest emit a diverse blend of 23 volatile organic compounds, including terpene and aromatic compounds, as well as amino-acid- and fatty-acid-derived compounds. The same volatile profile was observed (Figure S1) when closed and open flowers from *F. alnus* in Germany and Belgium, representing its native European range, were analyzed. In line with their role in pollinator attraction, the formation of floral VOCs frequently changes over flower development, with the highest levels being found in open flowers that are ready for pollination. Remarkably, of the 23 compounds observed with *F. alnus* flowers, only four were emitted in significantly higher quantities from open flowers compared to flower buds (Figure 6b). The higher emission rates of the aromatic compounds phenylethanol, ethyl benzoate, and ethyl salicylate, as well as the sesquiterpene α-farnesene, suggest a potential role of these compounds in the attraction of pollinators to *F. alnus* flowers. Although present in similar or even higher quantities than the four upregulated compounds (Figure 6a), the emission of many other *F. alnus* floral volatiles, including the monoterpenes α-pinene, β-pinene, camphene, D-limonene, β-ocimene, and β-linalool, as well as the aromatic compound benzyl benzoate, was not changed upon the opening of flowers (Figure 6b).

## 4. Discussion 

This study represents the first comprehensive investigation into the arthropod community associated with *F. alnus* conducted in North America. In total, 2845 specimens of insects and arachnids were systematically sampled, collectively constituting 563 distinct morphospecies. The abundance of arthropod orders was intricately linked to the phenological characteristics and seasonal variations observed during the survey. Notably, there were significant disparities in proportional representation among arthropod orders, being statistically different across the five designated collection dates, except for the other orders category.

Gassmann et al. [6] previously conducted a parallel inquiry into the insect fauna linked with *F. alnus* and *R. cathartica* in Europe, differing in their approach by targeting specialized insects with direct plant interactions, in contrast to our holistic collection strategy with limitations including the less intensive sampling of target groups such as stem-feeding or root-feeding insects. Their study revealed Lepidoptera as the predominant insect order, encompassing 22 distinct species across the two plant species, followed by Hemiptera (eight species), Diptera (four species), and Acarina (four species). However, it is worth noting that our findings diverge from this pattern, with Hemiptera emerging as the dominant order, specifically exemplified by 625 individuals of *P. carpinicola*, which was the dominant species; remarkably, 16.2% of these Hemiptera individuals were in the immature stages. Currently, there is extremely limited literature regarding *P. carpinicola*, and no detailed life history information is available; *P. carpinicola* is not known to feed on *F. alnus*, and our field observations did not reveal any conspicuous feeding damage attributable to this species. However, we observed *Carpinus betulus* (Betulaceae), the main host of *P. carpinicola*, in all study sites. This observation is consistent with that of another entomologist [26], who often observed *P. carpinicola* resting on beech trees, which are similarly a non-host species. Whether *P. carpinicola* feeds on *F. alnus* or not needs to be investigated further in the future.

*Drosophila suzukii* was the second most abundant species captured through net sweeping and was the sole species successfully reared from collected *F. alnus* fruits. Intriguingly, fruits from which *D. suzukii* emerged exhibited conspicuous signs of damage, with extensive fruit consumption and residual rotting, corroborating findings from Hauser et al. [27]. This suggests that *F. alnus* serves as a reproductive host of *D. suzukii* within forests with no agricultural fields nearby. Consequently, this suggests a potential negative impact on the dissemination and recruitment of *F. alnus*, particularly if the seeds prove non-viable when the fruits are infested by *D. suzukii*. It is pertinent to acknowledge that *D. suzukii* also exploits other forest flora, such as *Rubus allegheniensis*, *Prunus serotina*, and *Sambucus nigra*, as documented by Lee et al. [28]. Additionally, Grassi et al. [29] reported *D. suzukii* infesting *F. alnus* fruits in Italy. These interactions between *D. suzukii* and *F. alnus* could potentially influence avian consumption patterns, as birds might prefer insect-infested fruits due to their heightened protein content, as elucidated by Manzur and Courtney [30]. Our results further unveiled that adult *D. suzukii*’s flight activity and oviposition on *F. alnus* were exclusively tied to the full maturation of *F. alnus* fruits, signifying a clear attraction towards and utilization of mature fruits for reproduction. These findings are consistent with those of Kenis et al. [31], who documented that *D. suzukii* uses *F. alnus* in European countries. Moreover, Lee et al. [28] in Oregon identified *D. suzukii* infesting the fruits of *F. purshiana*, another *Frangula* species that is native to Western North America. If *F. alnus* inhabits near orchards, it can be used potentially as a trap plant for the monitoring of *D. suzukii*. 

Our survey revealed that there are numerous predatory arthropods indirectly associated with *F. alnus*, warranting particular attention because they are generalist carnivores [32]. Their pronounced presence in our samples indicates an abundant arthropod prey associated with *F. alnus*. The presence of such natural enemies can affect the overall diversity because they can inhabit and feed on prey insects on *F. alnus* throughout the year. Specifically, we observed significantly elevated arachnid abundance in August 2021, September 2021, and June 2022, when arthropod abundance was higher.

The arthropod community associated with *F. alnus* was highly dynamic, with substantial variations observed among different phenological stages (i.e., vegetative and reproductive stages). Both species richness and diversity exhibited a dependency on the sampling date, each dataset featuring at least one date with statistically distinct richness and diversity measures. Notably, the months of August and September 2021 exhibited a notable upsurge in arthropod abundance, coinciding with the peak foliage period when the plant provided substantial shade and refuge for insects traversing the adjacent roadways. These months were also characterized by heightened fruit availability. The patterns of richness and diversity were similarly influenced by the sampling date. July 2022 was marked by reduced insect and arachnid abundance and was not significantly abundant in any individual arthropod order. We attribute this phenomenon to the elevated temperatures experienced during sunny afternoons in July, which coincided with our insect collection efforts. 

While plant-herbivore interactions frequently involve VOCs with attractive and/or repellent activity, our analysis of *F. alnus* leaves revealed that these are not strongly scented and only emit comparatively small amounts of a few VOCs (Figure 5a). This might be one reason why only a limited number of herbivores were found associated with *F. alnus* in our study. It remains to be shown if this limited volatile emission is related to the relatively prominent cuticle, characteristic of *F. alnus* leaves, as recent reports have demonstrated a significant effect of the cuticle thickness and composition on the emission of volatile compounds [33,34]. However, it is noteworthy that the wounding of *F. alnus* leaves resulted in a significant increase in the emission of the terpenes β-ocimene and α-farnesene (Figure 5b). Many previous studies have reported the induction of β-ocimene and α-farnesene upon herbivory in many plant species [35,36,37]. Remarkably, α-farnesene is not only perceived in the antennal lobe of female *Helicoverpa assulta* (Lepidoptera: Noctuidae) but also found to have an inhibitory effect on their oviposition [35]. Moreover, several studies have shown that α-farnesene has an attractive effect on predators, such as anthocorid bugs (Hemiptera: Anthocoridae) [38] and parasitoids including *Telenomus podisi* (Hymenoptera: Platygastridae) and *Campoletis chlorideae* (Hymenoptera: Ichneumonidae) [35]. Further studies will be required to verify whether the observed α-farnesene emission of *F. alnus* in its invasive range also contributes to the attraction of parasitoids or predators such as the arachnids found in our arthropod survey. 

Insects are the only known pollinators of *F. alnus* [1], and since its flowers are self-incompatible and depend on cross-pollination, the interaction of *F. alnus* with pollinator insects is essential for the reproduction of *F. alnus* and the successful establishment and spread in its invasive range. It is important to note that the flowering period of *F. alnus* is unusually long, which suggests that it is likely pollinated by many different insect species [39]. While we only observed a relatively small number of potential pollinators that visited the *F. alnus* flowers, the consistently high fruit set indicated that a sufficient number of pollinator insects must have been visiting the *F. alnus* flowers. Of the four major *F. alnus* flower-visiting insects, *B. impatiens*, *A. pura*, and *R. recta* are well-known pollinators [40,41,42]. In contrast, *V. maculifrons* generally visit flowers to feed on nectar [43] and thus might not significantly contribute to the pollination of *F. alnus*. Our analysis of floral volatiles revealed that four VOCs (i.e., phenylethanol, ethyl benzoate, ethyl salicylate, and α-farnesene) are emitted from open *F. alnus* flowers at significantly higher rates, suggesting their involvement in the attraction of pollinators. These volatile compounds, particularly ethyl benzoate and ethyl salicylate, which are always found together, have previously been observed in the floral volatile profiles of other plant species, including strawberry (*Fragaria x ananassa*), wild tobacco (*Nicotiana attenuata*), lily (*Lilium* spp.), orange jessamine (*Murraya paniculata*), and *Viola tricolor* [44,45,46,47,48]. Remarkably, *Bombus terrestris* (Hymenoptera: Apidae) and *Apis mellifera* (Hymenoptera: Apidae) showed responses to phenylethanol, ethyl salicylate, and ethyl benzoate in an electroantennography (EAG) analysis [44]. The very strong response of *B. terrestris* towards ethyl benzoate reported in this recent study might explain our observation of *B. impatiens* visiting *F. alnus* flowers. An earlier EAG study also found a response of *Helicoverpa armigera* (Lepidoptera: Noctuidae) towards ethyl benzoate [49]. It should be considered that our survey of pollinator insects visiting *F. alnus* was restricted to daylight hours, and thus we cannot exclude that night-active insects such as moths might also visit *F. alnus* flowers and contribute to their pollination. In addition, some of the volatiles identified in this study can be used to develop attractants to increase the herbivory of *F. alnus*, but this will also require additional studies under controlled conditions to identify those VOCs that are perceived by and act attractive towards different herbivores.

Future studies may seek to identify the insect pollinators and birds associated with *F. alnus*. It is also unknown which bird species in North America may be more likely to increase the germination and overall success of *F. alnus* seeds. In addition, research on feeding damage by the four major insect species found in this study would give further insight as to how the insects can affect *F. alnus*. Finally, as this study found that *F. alnus* can be an excellent reproductive host of *D. suzukii,* future studies should seek to identify how this interaction between these two invasives (1) affects the germination of *F. alnus* seeds, (2) decreases the desirability of the fruit for the birds that disperse the seeds, (3) increases connectivity between the suitable ranges of *D. suzukii* within forests, and (4) is affected by the VOCs associated with *F. alnus* fruits. 

## 5. Conclusions

This research represents the first endeavor in the exploration of arthropods within the North American ecosystem that could serve as potential agents for the biological control of *F. alnus*, while also offering insights into the broader arthropod community associated with *F. alnus*. The results of this study revealed that many arthropods were directly and indirectly associated with *F. alnus,* and Hemiptera and Diptera were the dominant insect orders identified. The arthropod community on *F. alnus* changed significantly throughout the 2-year research period, indicating complex interactions among arthropods and *F. alnus*. However, no major herbivorous insects were found in this study that would cause significant damage to *F. alnus*. Although none of the insects found in this study could be used as a biological control agent immediately, our study found that a very diverse group of insects are utilizing *F. alnus* directly and indirectly. 

Our analysis demonstrated that *F. alnus* leaves are not strongly scented and, except for α-farnesene, only emit small amounts of a mixture of volatile compounds also commonly found in other plant species. Thus, it appears likely that not primarily volatile emission but rather the presence of anthraquinones with known antifeeding activity in *F. alnus* limit herbivore interactions in the invasive North American range, in contrast to its native European environment, where specialist herbivores are present. This study also reports on the volatile profile of *F. alnus* flowers, including a set of compounds specifically emitted from open flowers that might explain visitation by some of the pollinators that we observed. However, further studies are required to evaluate the visitation of *F. alnus* flowers by night-active insects and their contribution to pollination, as well as of florivores, which could potentially reduce the fruit set. Notably, *D. suzukii*, the second most frequently encountered species in our survey, was observed in both the net sweeping and fruit emergence collections. The utilization of *F. alnus* fruits by this invasive fly species accentuates the urgency of *F. alnus* management for the preservation of native berrying plants and crops.

## Figures and Tables

**Table 1:** The total number of arthropod orders associated with *F. alnus* collected in this study by using aerial net sweeping.

Order	August 2021	September 2021	May 2022	June 2022	July 2022	Total
Arachnida	119	161	31	115	16	442
Coleoptera	70	34	4	48	7	163
Diptera	165	225	154	81	10	635
Hemiptera	394	114	21	384	218	1131
Hymenoptera	130	63	33	30	19	275
Lepidoptera	33	9	21	22	5	90
Other	11	3	13	2	9	38
Psocoptera	11	54	1	5	0	71
Total	933	663	278	687	284	2845

## Data Availability

The data presented in this study are available on request from the corresponding author.

## Supplementary Materials

The following supporting information can be downloaded at: https://www.mdpi.com/2075-4450/14/12/913/s1, Figure S1: Characterization of the profile of volatile organic compounds emitted from *F. alnus* flowers collected in its native range in Germany (a) and Belgium (b). Volatiles were analyzed by GC/MS and total ion chromatograms are shown for flowers before (closed flowers) and after anthesis (open flowers). Compounds were identified based on their mass spectra and retention time; see Figure 6 for compound identity.
