# Supplementary material for: Arthropods Associated with Invasive Frangula alnus (Rosales: Rhamnaceae): Implications for Invasive Plant and Insect Management
Source: Insects. 2023 Nov 28;14(12):913. doi: 10.3390/insects14120913 (PMC10871088; doi:10.3390/insects14120913)
Supplement: Supplementary file 1 [file insects-14-00913-s001.zip › insects-2668146-supplementary.pdf]

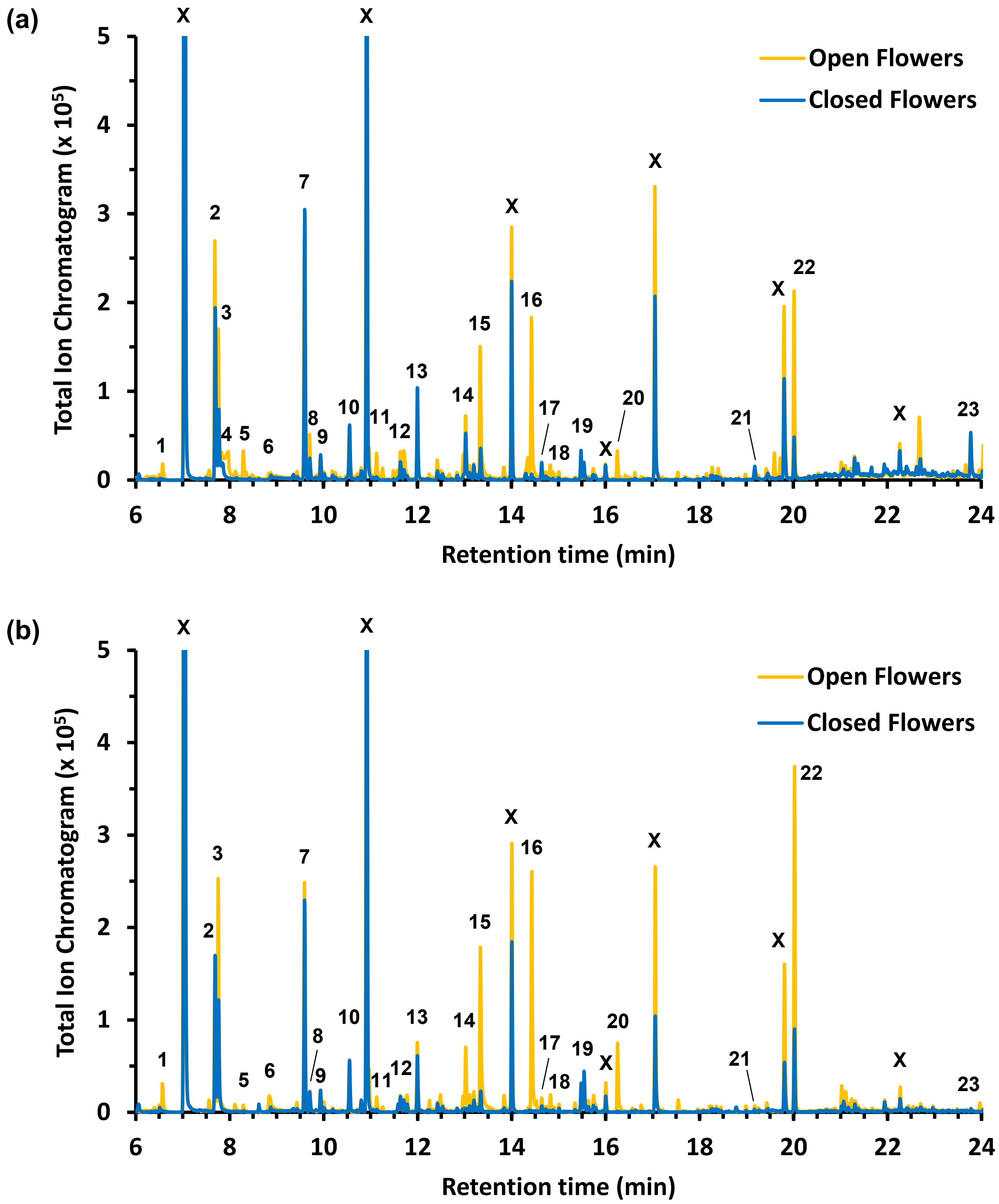

Figure S1: Characterization of the profile of volatile organic compounds emitted from *F. alnus* flowers collected in its native range in Germany (a) and Belgium (b). Volatiles were analyzed by GC/MS and total ion chromatograms are shown for flowers before (closed flowers) and after anthesis (open flowers). Compounds were identified based on their mass spectra and retention time, see Figure 6 for compound identity.
